# How Do Cyber Victimization and Low Core Self-Evaluations Interrelate in Predicting Adolescent Problematic Technology Use?

**DOI:** 10.3390/ijerph18063114

**Published:** 2021-03-18

**Authors:** María Angeles Peláez-Fernández, María Teresa Chamizo-Nieto, Lourdes Rey, Natalio Extremera

**Affiliations:** 1 Department of Social Psychology, Social Work, Social Anthropology and East Asian Studies, University of Málaga, 29071 Málaga, Spain; nextremera@uma.es; 2 Department of Personality, Assessment and Psychological Treatment, University of Málaga, 29071 Málaga, Spain; mtchamizo@uma.es (M.T.C.-N.); lrey@uma.es (L.R.)

**Keywords:** problematic technology use, cyber victimization, core self-evaluations, anxiety, stress, buffering effect

## Abstract

Research has demonstrated that cyber victimization is consistently associated with higher problem behaviors such as problematic technology use. However, little research has examined specific individual dispositions that can serve as a buffer in the link between cyber victimization and higher problematic uses of technology (i.e., problematic Internet, smartphone, and social media), such as core self-evaluations (CSE). A convenience sample of 1211 high school students, 657 females, 554 males, aged 12 to 18 (mean age = 13.74) completed measures of cyber victimization, CSE, and different problematic technology-related behaviors. Results of correlational analysis revealed significant associations between cyber victimization and all problematic uses of technology. Our findings also suggested that high CSE weakened the relationship between cyber victimization and two of the three problematic uses of technology. Consistent with social compensation theory, cyber victimization was concurrently linked to different problematic uses of technology. Low CSE also strengthened the link between cyber victimization and problems use of smartphones and social media and also showed a marginally significant interaction with cyber victimization in predicting problematic Internet use. Implications of these preliminary findings are discussed and avenues for further research are offered.

## 1. Introduction

### 1.1. Problematic Technology Usage and Cyberbullying

During the last two decades, new changes in the frequency and use of communication technologies have appeared. The advances and uses of the Internet, digital devices (e.g., smartphones) and applications are improving multiple areas of life (e.g., work, academic, personal, or social), facilitating easy individual access and sharing of information and resources, as well as interaction with other people on social networking sites. Adolescents, who face multiple changes in their stage of life, can take advantage of the benefits of these new technologies to foster social and family relationships, transfer knowledge and learn, or enjoy their leisure time [1]. Some theorists have linked the use of technologies with managing emotional and social deficits. For example, according to the social compensation hypothesis, adolescents showing shyness, loneliness, and social anxiety may use online communication to reduce their difficulties in face-to-face relationships, thus improving the quality of their social interactions and, as consequences, increasing their well-being [2,3].

Despite these potential benefits, the use of technology may become problematic in the adolescent population. A problematic use of technology here refers not only to spending an excessive amount of time using a smartphone, surfing the Internet, or interacting on social media, but also includes replacing or diminishing dedication to other important activities, such as academic performance, enjoyment of seeing friends face-to-face or performing offline leisure and cultural activities. [4]. A recent meta-analytic study [5] found an average prevalence of 23.3% of adolescents presenting a problematic use of smartphones, with prevalence ranging from 10% to 30% across studies. Besides, Loladze [6] in a systematic review has reported an average global prevalence of 13.1% adolescents with problematic use of Internet. Finally, regarding prevalence of adolescents with problematic social media use, a recent review [7] has found an average global prevalence of 7.38%, being the average prevalence rate in Spain 14.17%.

Inappropriate technology use may provide negative consequences for health, as well as potentially creating interpersonal problems (e.g., [8]). Specifically, previous studies have provided evidence that adolescents showing problematic Internet use had lower levels of well-being and self-concept and greater levels of impulsivity (e.g., [9,10]). Likewise, psychological maladjustment and mental health problems are typically associated with problematic smartphone use [5]. Similarly, with the use of social networking sites, a lower level of well-being and more frequent psychological complaints are often experienced by adolescents who engage in high problematic technology use [7].

Considering the negative impact caused by inappropriate use of technologies and electronic devices on adolescents’ quality of life and health, several authors have recently shown interest in examining the risk and protective factors involved in the development and maintenance of such use (e.g., [11,12]). Drawing on the compensatory internet use theory [13], motivations and their antecedents are considered as important factors for the use of online technology. This theoretical approach suggest that, when experiencing stressful situations and psychological problems, adolescents may use the Internet to compensate for these negative feelings, which increases the risk of developing a pattern of problematic use [13]. One of the situations that causes high levels of stress in adolescent population is having experienced cyberbullying behaviors [14]. Likewise, previous studies have found that suffering cyber victimization constituted one of the key predictors for the development of problematic technology use (e.g., [15,16,17]).

Cyberbullying is an intentional and repetitive behavior of aggression toward others carried out throughout cyberspace or using electronic devices [18]. This behavior shows specific characteristics, by which it can be differentiated from traditional bullying, as it is quicker, wider, and allows constant diffusion and the possibility for the aggressor to maintain anonymity [19]. It is thus more difficult for victims to defend themselves. Despite the different prevalence of cyberbullying by country (e.g., [20,21]), this problem is present all over the world. Average global prevalence of reported victimization by cyber-bullying is 11.9 per 100 children for 13 yr.-old boys, and 11.3 for 15 yr.-old boys. Mean global prevalence for girls is slightly higher: 13.9 for 13 yr.-old girls and 12.7 for 15 yr.-old girls [21]. A recent cross-national study carried out with European students aged 7–19 years from eight countries by Sorrentino et al. [22], pointed out that one in four had suffered from cyberbullying during the past twelve months.

A bulk of the research provides evidence about the important negative impact on adolescents’ psychological adjustment and well-being among those who suffer or have suffered cyber victimization. Among the negative consequences, previous studies have found lower levels of life satisfaction and self-concept; more depressive symptoms [23,24]; higher levels of social anxiety, stress, and loneliness [24]; and higher suicide risk [25,26]. Moreover, suffering from cyber victimization is related to an increased likelihood of engaging in problem behaviors such as substance use, gambling or problematic Internet, smartphone, and social media use (e.g., [21,27,28,29]).

Considering the negative impact of cyber victimization and inappropriate technology use on adolescents’ well-being, as well as the strong link between both issues, some researchers have examined the role of protective factors to reduce the likelihood of that adolescents who have experienced cyber victimization will develop problematic technology use (e.g., [17]). In particular, one of the potential protective factors that could buffer the link between cyber victimization and problematic technology use among adolescents is core self-evaluation (CSE) [30,31]. 

### 1.2. Core Self-Evaluations

The CSE construct is defined as a higher-order trait comprising the variance shared by four highly related and well-established personality traits: self-esteem, generalized self-efficacy, locus of control, and emotional stability [32]. Self-esteem refers to the overall value that one grants oneself as a person [33]. Generalized self-efficacy is a global evaluation of how well one can function across diverse tasks and situations [34]. Emotional stability (often considered in opposition to neuroticism) is the propensity to have a positive cognitive/interpretive style and to focus on positive (instead of negative) facets of the self [35]. Finally, locus of control refers to a belief system in which individuals internalize the causes and motives of events in their life, making them see events as being (or not) contingent on their own behavior [36]. In sum, CSE reflects how worthy, capable, and effective a person feels and thus represents the basic and fundamental evaluations that individuals make about themselves and their own functioning in the environment [37]. Therefore, individuals high in CSE evaluate themselves in a consistently positive way across situations [38].

In terms of coping with conflicting situations, adolescents with high CSE should be better able to manage adversity, as they feel themselves highly qualified and able to handle the situation and worthy to warrant the derived rewards [38]. Along these lines, the meta-analysis by Kammeyer-Muller et al. [39] found that CSE was associated with a lower frequency of experiencing stressful circumstances and that the relationship between the experience of stress and the feeling of strain was weaker for those with positive CSE. Both findings suggest that individuals with high CSE are capable of coping with stressful situations without enduring severe physical and emotional exhaustion. Thus, this meta-analysis supplies evidence that those with high CSE tend to avoid coping in ineffective ways. 

In the case of cyberbullying and cyber victimization, some recent studies have found a significant association among low levels of CSE and higher involvement in cyberbullying behaviors and cyberbullying victimization [40]. Several studies also support the role of specific CSE components as protective factors. For example, high self-esteem [41,42,43,44,45,46,47], self-control [46,48,49], and self-efficacy [44,46,50] have been identified as protective factors for bullying victimization among middle and high school adolescents.

In terms of problematic technology use, there has also recent been empirical evidence that suggests adolescents’ appraisals of their fundamental self-worth and capabilities might constitute an underlying dimension that might limit the development of potentially addictive online activities. For example, prior studies have found that core self-evaluation was a precursor of social networking site addiction among Chinese adolescents [51]. CSE and levels of Internet addiction have also shown significant and negative associations in a sample of college students [31]. Finally, some preliminary findings have found the potential influence of specific dimensions of CSE on problematic technology use. For example, self-esteem [52] and self-regulation [11] were significant protective factors against problematic technological use; and neuroticism (as opposite to emotional stability) has been associated to addictive and problematic technology use [10,53,54,55] among an adolescent population. Therefore, it is expected that adolescents with high CSE will be better able to handle technology use in a responsible and moderate way, even if they have suffered victimization. 

In line with the social compensation hypothesis [2,3], these studies suggest that peer victimization might affect adolescents’ self-esteem and self-control, increasing negative symptoms and loneliness, thus leading to the misuse of technology as a means of alleviating the decline into negative moods. It has been suggested that those adolescents with high levels of CSE suffering from cyberbullies’ behaviors would report less frequency and problematic use of technology. In other words, it is expected that these dispositions act as a buffer between victimization and problematic technology use.

Although some research has explored the role of CSE on problematic technology use and cyber victimization independently, this has not been comprehensively investigated, and researchers have specifically called for further research examining the buffering effects of CSE on different problematic technology (i.e., Internet, smartphone, and social media) use, particularly in high-risk samples such as adolescents who may have been cyber victimized by their peers. Examining this potential role is crucial in understanding the mechanisms underlying the association between problematic technology use and cyber victimization. Thus, the aim of this study is to bridge the research gap by testing the role of CSE in the relationship between cyber victimization and problematic technology use among adolescents. This knowledge might be fruitful in developing preventive programs that promote CSE-related personal resources among adolescents.

### 1.3. The Current Study

To deepen the knowledge of the link between cyber victimization and problematic technology use and to examine the role of CSE in this relationship, the current study had two objectives. First, the relationship between cyber victimization and different kinds of problematic technology use (i.e., Internet, smartphone, and social media) was analyzed. In line with previous research (e.g., [16,17]), we expect to find that the greater the extent of the cyber victimization suffered, the more problematic the Internet, smartphone, and social media use by the adolescent population. Second, the moderation effect will be analyzed. According to previous work (e.g., [36,38,39]), we expect to find that high CSE would act as a buffer weakening the link between cyber victimization and different types of problematic technology use. Specifically, we expect that the adolescents high in CSE who suffer cyber-victimization will report less problematic smartphone use and social media use; and that those low in CSE will report more problematic smartphone use and social media use. 

## 2. Materials and Methods

### 2.1. Participants and Procedures

A sample of 1211 adolescents belonging to five education centers in the South of Spain participated by filling out several questionnaires anonymously and voluntarily. Of the respondents, 657 identified as female and 554 as male, ranging in age from 12 to 18 years (*M_age_* = 13.74, *SD* = 1.33). Most were of Spanish nationality (97%; 13 did not answer the question) and attended school in Grades 7 to 10.

The research was conducted in line with the Declaration of Helsinki [56] and was approved by the Ethical Committee of the University of Malaga (62-2016-H). Initially, 12 education centers were contacted to participate in the study, with an acceptance ratio of 41.67% (only 5 centers agreed to collaborate). Directors and board members of the five education centers were informed about the objectives and procedure of the research. A consent form was signed by every director. Later, students’ legal tutors received information about the study and agreed to participate, signing a consent form (four centers) or did not clearly refuse to content to participation by the adolescents (one center). Two researchers gave instructions and answered any questions while students were filling out the questionnaires. 

### 2.2. Measures

Before filling in the questionnaires, some demographic variables were requested, such as age, gender, nationality, and study grades. Cyber victimization was measured using the cyberbullying victimization subscale of the Spanish version of the European Cyberbullying Intervention Project Questionnaire (ECIPQ; [57], originally developed by Del Rey et al. [58]). This subscale assesses the frequency of cyberbullying experiences suffered over the last two months. It comprises 11 items, which are answered using a five-point Likert scale, with a range from 0 (never) to 4 (multiple times a week). An example item is “Someone threatened me through texts or online messages.” The Cronbach’s α obtained in the current study was 0.85.

Problematic use of the Internet was measured using the Spanish version of the Addiction Internet Test (IAT; [59], originally developed by Young [60]). This test comprises 20 items assessing how Internet use over the last twelve months, as well as at the starting grade, begins to affect to several areas (i.e., habits, social, productivity, feelings, and sleep pattern); a sample item is “How often do you lose sleep due to being online?” Each item is answered using a five-point Likert scale, with 1 being “rare” and 5 “always”. The reliability in the present study was 0.83.

The problematic use of smartphones was assessed by the Spanish short version of the Smartphone Addiction Scale (SAS-SV; [61], originally developed by Kwon et al. [62]). This scale assesses the use of mobile smartphones over the last year and if this use may indicate a problem. It comprises 10 items, which are answered using a six-point Likert scale, with 1 being “strongly disagree” and 6 “strongly agree.” An example item is “Won’t be able to stand not having a smartphone.” In the current study, the Cronbach’s α was 0.85.

The problematic use of social media was measured by the Social Media Addiction Questionnaire (SMAQ; [63]). This questionnaire comprises 8 items assessing the use of social media such as Facebook, Twitter, and Snapchat among others; a sample item is “The thought of not being able to access social media makes me feel distressed.” The items are answered using a seven-point Likert scale, with a range from one (strongly disagree) to seven (strongly agree). In the current study, a back-translation method was performed to create a Spanish version, obtaining a Cronbach’s α of 0.87.

The CSEs were assessed by the Spanish version of the Core Self-Evaluations Scale (CSES; [64], originally developed by Judge et al. [37]). This scale assesses four specific traits overall, which underlie the self-evaluations (i.e., neuroticism, locus of control, self-esteem, and generalized self-efficacy). It comprises 12 items, which are answered using a five-point Likert scale, with 1 being “strongly disagree” and 5 “strongly agree”. An example item is “I am confident I get the success I deserve in life.” The reliability obtained in this study was 0.74.

### 2.3. Data Analysis

The data analyses were performed using SPSS v23 and the PROCESS macro [65]. First, all missing items values were imputed using the expectation-maximization (EM) method with SPSS v23. Later, descriptive statistics, internal consistency, and Pearson correlation analysis among the study variables were calculated. We then examined the interaction between cyber victimization and core self-evaluations for problematic use of each of the new technologies (i.e., Internet, smartphones, and social media). We examined the moderating aspects using Hayes’ PROCESS macro (Model 1) [65].

## 3. Results

### 3.1. Descriptive Analyses

Means, standard deviations, bivariate correlations, and reliability for all study variables are presented in Table 1. As expected, CSE was negatively correlated with problematic technology use for all three technologies considered (i.e., Internet use, smartphones, and social media) and cyber victimization. Thus, cyber victimization was positively associated with all three types of problematic technology use.

### 3.2. Gender Differences

Difference tests were performed in order to analyze the potential gender differences in CSE, cyber victimization and problematic technology use (Internet, social media and smartphone). A one-way ANOVA analysis revealed significant gender differences for the following variables: CSE (*M* = 3.32 for females and *M* = 3.53 for males; *p* < 0.01), problematic smartphone use (*M* = 2.66 for females and *M* = 2.17 for males; *p* < 0.01) and problematic social media use (*M* = 25.40 for females and *M* = 18.66 for males; *p* < 0.01). Finally, no significant differences were found for cyber victimization (*M* = 0.25 for females and *M* = 0.25 for males; *p* = 0.87 and problematic Internet use (*M* = 2.08 for females and *M* = 2.04 for males; *p* = 0.19).

### 3.3. Moderator Analyses

To test the hypothesis that CSE would moderate the relationship between cyber victimization and problematic technology use, controlling for age and gender because of significant differences, the SPSS macro PROCESS was used. This macro runs a series of OLS (ordinary least squares) regressions with the centered product term representing the interaction of cyber victimization × CSE as a predictor of the three types of problematic technology use. We computed the bootstrapped bias-corrected 95% confidence intervals by taking 5000 bootstrapped samples [65]. All interactions were further examined using the simple slope analysis procedure as implemented in the PROCESS macro (see Table 2).

#### 3.3.1. Problematic Internet Use

The full prediction model, including covariates, main variables, and the interaction term, accounted for 18% of the variance observed in problematic Internet use (R^2^adj = 0.18, F(1205) = 54.45, *p* < 0.01). Age (*p* < 0.05) was found to explain significant variance in problematic Internet use. In addition, the main effect of cyber victimization (*p* < 0.001) was significant in explaining variance in problematic Internet use. Finally, a marginally significant interaction effect between cyber victimization and CSE (*p* < 0.10) was found to account for a marginally significant amount of additional variance in problematic Internet use (ΔR^2^ = 0.002), after partialing out the variance accounted for by covariates, cyber victimization, and CSE.

#### 3.3.2. Problematic Smartphone Use

**Figure 1:**
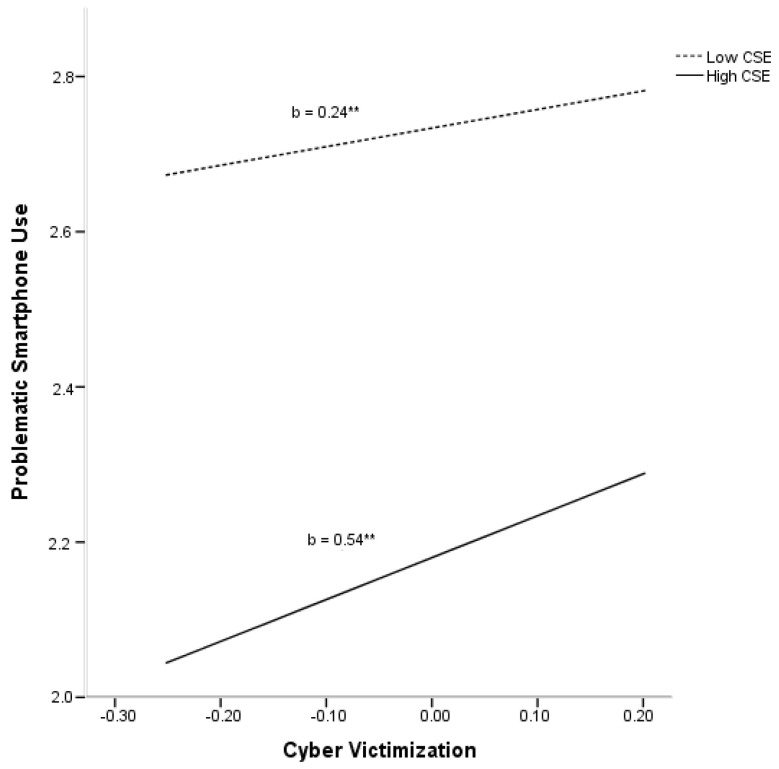
Relationship of cyber victimization and core self-evaluations (CSE) for predicting problematic smartphone use. ** *p* < 0.01.

The final model, including covariates, main variables, and the interaction term, accounted for 22% of the variance observed in problematic smartphone use (R^2^adj = 0.22, F(1205) = 67.13, *p* < 0.01). Age (*p* < 0.01) and gender (*p* < 0.01) were found to explain significant variance in problematic smartphone use. In addition, a significant main effect was found for CSE (*p* < 0.01). Finally, a significant interaction effect between cyber victimization and CSE (*p* < 0.05) was found to account significantly for additional variance in problematic smartphone use (ΔR^2^ = 0.004), after partialing out the variances accounted for by covariates and both predictors (see Figure 1).

#### 3.3.3. Problematic Social Media Use

**Figure 2:**
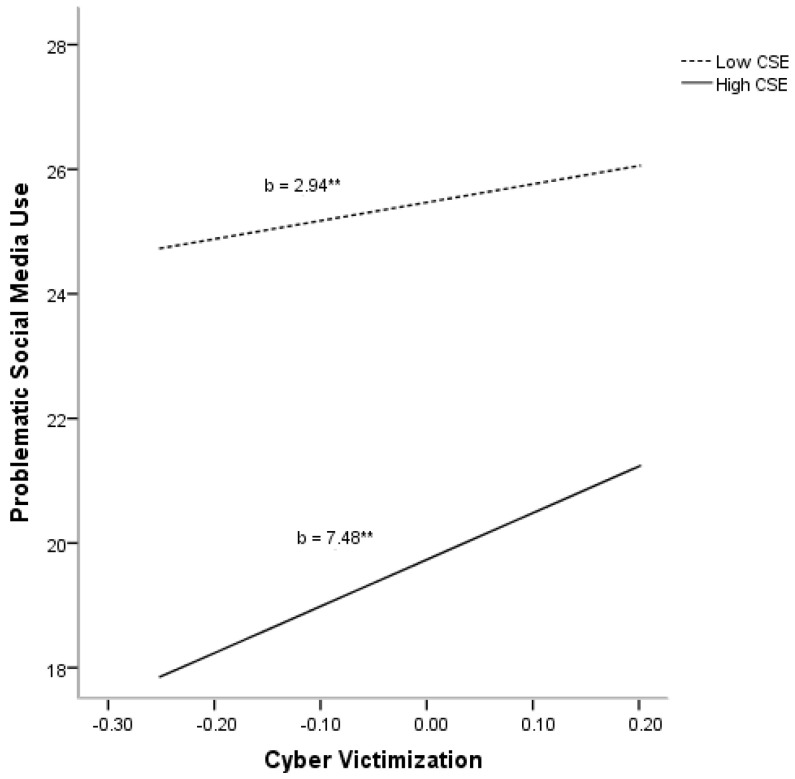
Relationship of cyber victimization and core self-evaluations (CSE) for predicting problematic social media use. ** *p* < 0.01.

The final model, which also included covariates, main variables, and the interaction effect, accounted for 27% of the variance in problematic social media use (R^2^adj = 0.27, F(1205) = 87.39, *p* < 0.01). Age (*p* < 0.01) and gender (*p* < 0.01) were found to significantly explain significant variance in problematic social media use. Moreover, significant main effects were found for both cyber victimization (*p* < 0.01) and CSE (*p* < 0.01). Finally, a significant interaction between cyber victimization and CSE (*p* < 0.01) was found to account significantly for additional variance in problematic social media use (ΔR^2^ = 0.008), after partialing out the variances accounted for by covariates, cyber victimization, and CSE (see Figure 2).

## 4. Discussion

The objective of this study was to examine whether CSE played a buffering role in the relationship between cyber victimization and problematic technology use among adolescents. This study therefore examined the relationship between cyber victimization and three types of problematic technology use (i.e., Internet, smartphone, and social media). As mentioned earlier, we expected to find that the greater the cyber victimization suffered, the greater the problematic Internet, smartphone, and social media use by a Spanish adolescent sample. Moreover, this study analyzed whether CSE would act as a moderator weakening the link between cyber victimization and every type of problematic technology use.

Regarding the correlation analyses, our findings supported our expectations in terms of establishing a significant and negative relationship between CSE and problematic technology use and cyber victimization. In addition, and in line with previous research (e.g., [15,16,66]), cyber victimization and problematic technology use were found to be significantly and positively related.

The results regarding gender differences were also identified in our study and indicated that males scored higher than females on CSE. This is consistent with previous studies that have found that males reported higher scores than females on CSE [67,68]. On the other hand, females scored higher than males in problematic smartphone and social media use. In this vein, a bunk of studies indicates that virtually, females have higher levels of dependence and problematic use than males [69,70,71]. Female smartphone and social media use are mainly related to social contact and to create or maintain interpersonal relationships [70,72].

Regarding the moderator role of CSE in the link between cyber victimization and problematic technology use, our results showed that the interaction between cyber victimization and CSE significantly improved the prediction of problematic smartphone and problematic social media use beyond the separate main effects of these constructs. The full model for problematic smartphone and social media use explained a higher proportion of the variance than was the case with the full model for problematic Internet use, where the variance explained was only marginally significant. In accordance with compensatory Internet use theory [13] and the social compensation hypothesis [2,3], the relationship between cyber victimization and problematic technology use may be due to victimized adolescents going online to distract themselves from harmful cyberbullying actions or to alleviate internalizing or externalizing problems; this can sometimes lead to both problematic technology use and lasting negative school and mental health issues (e.g., [24]). Therefore, cyber victim adolescents might try to mitigate the negative impact by technology use to improve their well-being [2,3]. Nevertheless, this tendency to use technology to alleviate negative feelings associated with having received cyberbullying actions may increase the likelihood of developing a maladaptive Internet, smartphone, or social media use habit.

Beyond the main effects, our findings suggest new potential ways in which CSE may, in combination with psychosocial factors such as having received cyberbullying actions, contribute in the prediction of problematic use of technology. Educators and clinicians may also seek to assess and explore for deficits in coping resources among adolescents with problematic use of technology, as this situation may contribute to cyber victimization and, subsequently, to more serious socio-emotional consequences (i.e., suicidal ideation, [25,26]).

The mechanisms through which CSE might mitigate the link between cyber victimization and problematic technology use may be diverse. In line with compensatory Internet use, CSE might act as a buffer mechanism between victimization and problematic technology use. That is, adolescents high in these dispositions might be better able to handle the stressful dysphoric moods caused by receiving peer cyber bullying, and thus less susceptible to compensation through problematic technology use. Another plausible explanation is that, theoretically, CSE may facilitate adolescents’ ability to cope with stressful situations such as cyber victimization without suffering severe negative and deleterious moods [38,39]; however, cyber victimized adolescents are more likely to rely on technology use to compensate for low self-worth [39]. Consistent with this perspective, adolescents with deficits in cognitive emotional resources might tend to interpret virtual peer conflicts as being more threatening than challenging, and also to use non-productive coping strategies such as problematic online social activities, thus leading them to endure deleterious psychological symptoms [39]. High CSE might, however, affect the way adolescents deal with online social events, inducing them to manage the received cyber victimization actions more effectively and thus experience fewer and less intense symptoms and adverse stress reactions [39]. Further research with prospective designs might help to clarify these potential mechanisms.

Several limitations should be considered in the current research. First, our research design was cross-sectional and based on self-reported measures, which precludes any causal inference and could be biased by social desirability. Further longitudinal studies using other types of assessment (i.e., interview techniques or teacher/parent reports) would contribute to the generalizability of our findings and clarifying the direction of the effects. In addition, our study was conducted with a community sample of adolescents; the results might thus not be generalizable to the clinical population. Future studies testing this moderating model in clinical samples are needed.

Concerning the practical implications, our findings suggest that educators and clinicians working with adolescents with higher levels of problematic technology use should assess both for potential deficits in personal dispositions such as CSE as well as levels of problematic technology use to identify those who might be at higher risk of experiencing negative consequences associated with cyber victimization. In addition, because personal resources are crucial to reducing the link between problematic technology use and cyberbullying and to improving the ability to cope with adversity and social conflicts, education curricula should include assessment for deficits in these resources and incorporate prevention and intervention programs aimed at helping at-risk peer cyber victimized adolescents to develop a sense of competency and capability about themselves, thus increasing their potential coping options and capacity to respond in advance of a potential cyberbullying event.

## 5. Conclusions

In conclusion, our findings support compensatory Internet use theory, which suggests that the negative impact of cyber victimization may boost problematic technology use as a strategy to mitigate the negative feelings derived from received cyberbullying actions. Understanding the personal resources that mitigate the link between cyber victimization and problematic technology use may help educators and clinicians to remedy deficits in self-worth involved in the early manifestation of cyber victimization to prevent or modify its form before it results in a worsening cycle of future negative symptoms, maladaptive reactions, and mental health issues.

## Figures and Tables

**Table 1:** Means, standard deviations, reliabilities and correlations between study variables.

	1	2	3	4	5
1. Core Self-Evaluations	-				
2. Problematic Internet Use	−0.37 ***	-			
3. Problematic Smartphone Use	−0.37 ***	0.65 ***	-		
4. Problematic Social Media Use	−0.38 ***	0.59 ***	0.78 ***	-	
5. Cyber Victimization	−0.26 ***	0.30 ***	0.23 ***	0.25 ***	-
M	3.42	2.07	2.44	22.33	0.25
SD	0.59	0.57	0.99	10.66	0.42
Alpha	0.74	0.83	0.85	0.87	0.85

Note. N = 1211. *** *p* < 0.001.

**Table 2:** Tested moderation models with problematic Internet use, problematic smartphone use and problematic social media use outcomes predicted by cyber victimization, core self-evaluation (CSE) and multiplicative interaction terms.

	B	SE b	R^2^	Δ R^2^	95% CI
Model 1. Problematic Internet Use			0.18 **		
Constant	1.786 **	0.165			1.463 to 2.108
Age	0.024 *	0.011			0.002 to 0.047
Gender	−0.026	0.030			−0.086 to 0.033
Cyber Victimization	0.318 **	0.042			0.236 to 0.041
CSE	−0.297	0.027			−0.350 to −0.245
Cyber Victimization x CSE	0.110 ^1^	0.060		0.002 ^1^	−0.008 to 0.228
Model 2. Problematic Smartphone Use			0.22 **		
Constant	0.435	0.281			−0.117 to 0.987
Age	0.104 **	0.020			0.065 to 0.142
Gender	0.385 **	0.052			0.283 to 0.486
Cyber Victimization	0.391	0.072			0.250 to 0.532
CSE	−0.474 **	0.046			−0.564 to −0.384
Cyber Victimization x CSE	0.257 *	0.103		0.004 *	0.056 to 0.459
Model 3. Problematic Social Media Use			0.27 **		
Constant	−0.646	2.928			−6.391 to 5.098
Age	1.051 **	0.204			0.651 to 1.451
Gender	5.687 **	0.538			4.631 to 6.743
Cyber Victimization	5.230 **	0.748			3.762 to 6.698
CSE	−4.915 **	0.476			−5.848 to −3.981
Cyber Victimization x CSE	3.895 **	1.071		0.008 **	1.795 to 5.996

Note. B = unstandardized beta; SE b = standard error of beta coefficients; R^2^ = R-squared; Δ R^2^ = incremental R-squared; 95% CI = 95% confidence interval. ^1^ *p* < 0.10; * *p* < 0.05; ** *p* < 0.01.

## References

[1] Ballesteros J.C., Picazo L. (2018). Las TIC y su Influencia en la Socialización de Adolescentes.

[2] Valkenburg P.M., Peter J. (2009). Social consequences of the internet for adolescents: A decade of research. Curr. Dir. Psychol. Sci..

[3] Valkenburg P.M., Peter J. (2011). Online communication among adolescents: An integrated model of its attraction, opportunities, and risks. J. Adolesc. Health.

[4] Díaz-Vicario A., Mercader C., Gairín J. (2019). Uso problemático de las TIC en adolescentes (Problematic ICT use in adolescents). Rev. Electron. Investig. Educ..

[5] Sohn S., Rees P., Wildridge B., Kalk N.J., Carter B. (2019). Prevalence of problematic smartphone usage and associated mental health outcomes amongst children and young people: A systematic review, meta-analysis and GRADE of the evidence. Bmc Psychiatry.

[6] Loladze T. (2020). Adolescents problematic internet use: Family factors systematic review. Transl. Clin. Med. Georgian Med. J..

[7] Boer M., van den Eijnden R.J.J., Boniel-Nissim M., Wong S.-L., Inchley J.C., Badura P., Craig W.M., Gobina I., Kleszczewska D., Klanscek H.J. (2020). Adolescents’ intense and problematic social media use and their well-being in 29 countries. J. Adolesc. Health.

[8] Chassiakos Y.R., Radesky J., Christakis D., Moreno M.A., Cross C., Hill D., Ameenuddin N., Hutchinson J., Boyd R., Mendelson R. (2016). Children and adolescents and digital media. Pediatrics.

[9] Machimbarrena J.M., González-Cabrera J., Ortega-Barón J., Beranuy-Fargues M., Álvarez-Bardón A., Tejero B. (2019). Profiles of problematic internet use and its impact on adolescents’ health-related quality of life. Int. J. Environ. Res. Public Health.

[10] Malo-Cerrato S., Martín-Perpiñá M.-M., Viñas-Poch F. (2018). Excessive use of social networks: Psychosocial profile of Spanish adolescents. Comunicar.

[11] Kiss H., Fitzpatrick K.M., Piko B.F. (2020). The digital divide: Risk and protective factors and the differences in problematic use of digital devices among Hungarian youth. Child. Youth Serv. Rev..

[12] Martín-Perpiñá M.-M., Viñas Poch F., Malo Cerrato S. (2019). Personality and social context factors associated to self-reported excessive use of information and communication technology (ICT) on a sample of Spanish adolescents. Front. Psychol..

[13] Kardefelt-Winther D. (2014). A conceptual and methodological critique of internet addiction research: Towards a model of compensatory internet use. Comput. Hum. Behav..

[14] Caravita S.C.S., Colombo B., Stefanelli S., Zigliani R. (2016). Emotional, psychophysiological and behavioral responses elicited by the exposition to cyberbullying situations: Two experimental studies. Psicol. Educ..

[15] Boniel-Nissim M., Sasson H. (2018). Bullying victimization and poor relationships with parents as risk factors of problematic internet use in adolescence. Comput. Hum. Behav..

[16] Gül H., Fırat S., Sertçelik M., Gül A., Gürel Y., Kılıç B.G. (2019). Cyberbullying among a clinical adolescent sample in Turkey: Effects of problematic smartphone use, psychiatric symptoms, and emotion regulation difficulties. Psychiatry Clin. Psychopharmacol..

[17] Wang Z., Xie Q., Xin M., Wei C., Yu C., Zhen S., Liu S., Wang J., Zhang W. (2020). Cybervictimization, depression, and adolescent internet addiction: The moderating effect of prosocial peer affiliation. Front. Psychol..

[18] Smith P.K., Mahdavi J., Carvalho M., Fisher S., Russell S., Tippett N. (2008). Cyberbullying: Its nature and impact in secondary school pupils. J. Child. Psychol. Psychiatry Allied Discip..

[19] Kwan G.C.E., Skoric M.M. (2013). Facebook bullying: An extension of battles in school. Comput. Hum. Behav..

[20] Lozano-Blasco R., Cortés-Pascual A., Latorre-Martínez M.P. (2020). Being a cybervictim and a cyberbully—The duality of cyberbullying: A meta-analysis. Comput. Hum. Behav..

[21] Craig W., Boniel-Nissim M., King N., Walsh S.D., Boer M., Donnelly P.D., Harel-Fisch Y., Malinowska-Cieślik M., Gaspar de Matos M., Cosma A. (2020). Social media use and cyber-bullying: A cross-national analysis of young people in 42 countries. J. Adolesc. Health.

[22] Sorrentino A., Baldry A.C., Farrington D.P., Blaya C. (2019). Epidemiology of cyberbullying across europe: Differences between countries and genders. Educ. Sci. Theory Pract..

[23] Estévez J.F., Cañas E., Estévez E. (2020). The impact of cybervictimization on psychological adjustment in adolescence: Analyzing the role of emotional intelligence. Int. J. Environ. Res. Public Health.

[24] Estévez E., Estévez J.F., Segura L., Suárez C. (2019). The influence of bullying and cyberbullying in the psychological adjustment of victims and aggressors in adolescence. Int. J. Environ. Res. Public Health.

[25] Chang Q., Xing J., Ho R.T.H., Yip P.S.F. (2019). Cyberbullying and suicide ideation among Hong Kong adolescents: The mitigating effects of life satisfaction with family, classmates and academic results. Psychiatry Res..

[26] Perret L.C., Orri M., Boivin M., Ouellet-Morin I., Denault A.S., Côté S.M., Tremblay R.E., Renaud J., Turecki G., Geoffroy M.C. (2020). Cybervictimization in adolescence and its association with subsequent suicidal ideation/attempt beyond face-to-face victimization: A longitudinal population-based study. J. Child. Psychol. Psychiatry.

[27] Zhu Y., Li W., O’Brien J.E., Liu T. (2019). Parent–child attachment moderates the associations between cyberbullying victimization and adolescents’ health/mental health problems: An exploration of cyberbullying victimization among Chinese adolescents. J. Interpers. Violence.

[28] Yoon Y., Lee J.O., Cho J., Bello M.S., Khoddam R., Riggs N.R., Leventhal A.M. (2019). Association of cyberbullying involvement with subsequent substance use among adolescents. J. Adolesc. Health.

[29] Yudes C., Baridon-Chauvie D., González-Cabrera J. (2018). Cyberbullying and problematic Internet use in Colombia, Uruguay and Spain: Cross-cultural study. Comunicar.

[30] Judge T.A., Van Vianen A.E.M., De Pater I.E. (2004). Emotional stability, core self-evaluations, and job outcomes: A review of the evidence and an agenda for future research. Hum. Perform..

[31] Geng J., Han L., Gao F., Jou M., Huang C.C. (2018). Internet addiction and procrastination among Chinese young adults: A moderated mediation model. Comput. Hum. Behav..

[32] Judge T.A., Locke E.A., Durham C.C. (1997). The dispositional causes of job satisfaction: A core evaluations approach. Res. Organ. Behav..

[33] Harter S., Sternberg R.J., Kolligian J. (1990). Causes, correlates, and the functional role of global self-worth: A life span perspective. Competence Considered.

[34] Locke E.A., McClear K., Knight D. (1996). Self esteem and work. Int. Rev. Ind. Organ. Psychol..

[35] Watson D. (2000). Mood and Temperament.

[36] Rotter J.B. (1996). Generalized expectancies for internal versus external control of reinforcement. Psychol. Monogr. Gen. Appl..

[37] Judge T.A., Erez A., Bono J.E., Thoresen C.J. (2003). The core self-evaluations scale: Development of a measure. Pers. Psychol..

[38] Elliott D.C., Kaliski P., Burrus J., Roberts R.D., Prince-Embury S., Saklofske D.H. (2013). Exploring adolescent resilience through the lens of core self-evaluations. Resilience in Children, Adolescents, and Adults: Translating Research into Practice.

[39] Kammeyer-Mueller J.D., Judge T.A., Scott B.A. (2009). The role of core self-evaluations in the coping process. J. Appl. Psychol..

[40] Yudes C., Rey L., Extremera N. (2020). Predictive factors of cyberbullying perpetration amongst Spanish adolescents. Int. J. Environ. Res. Public Health.

[41] Álvarez-García D., Núñez Pérez J.C., Dobarro González A., Rodríguez Pérez C. (2015). Risk factors associated with cybervictimization in adolescence. Int. J. Clin. Health Psychol..

[42] Bayraktar F., Machackova H., Dedkova L., Cerna A., Ševčíková A. (2015). Cyberbullying: The discriminant factors among cyberbullies, cybervictims, and cyberbully-victims in a Czech adolescent sample. J. Interpers. Violence.

[43] Brewer G., Kerslake J. (2015). Cyberbullying, self-esteem, empathy and loneliness. Comput. Hum. Behav..

[44] Chen L., Ho S.S., Lwin M.O. (2017). A meta-analysis of factors predicting cyberbullying perpetration and victimization: From the social cognitive and media effects approach. New Media Soc..

[45] Kowalski R.M., Limber S.P. (2013). Psychological, physical, and academic correlates of cyberbullying and traditional bullying. J. Adolesc. Health.

[46] Kowalski R.M., Limber S.P., McCord A. (2019). A developmental approach to cyberbullying: Prevalence and protective factors. Aggress. Violent Behav..

[47] Zych I., Farrington D.P., Ttofi M.M. (2019). Protective factors against bullying and cyberbullying: A systematic review of meta-analyses. Aggress. Violent Behav..

[48] Baldry A.C., Farrington D.P., Sorrentino A. (2015). A narrative review and conceptual framework for research on risk of cyberbullying and cybervictimization: The risk and needs assessment approach. Aggress. Violent Behav..

[49] Vazsonyi A.T., Machackova H., Sevcikova A., Smahel D., Cerna A. (2012). Cyberbullying in context: Direct and indirect effects by low self-control across 25 European countries. Eur. J. Dev. Psychol..

[50] Solomontos-Kountouri O., Tsagkaridis K., Gradinger P., Strohmeier D. (2017). Academic, socio-emotional and demographic characteristics of adolescents involved in traditional bullying, cyberbullying, or both: Looking at variables and persons. Int. J. Dev. Sci..

[51] He D., Liu Q.-Q., Shen X. (2021). Parental conflict and social networking sites addiction in Chinese adolescents: The multiple mediating role of core self-evaluation and loneliness. Child. Youth Serv. Rev..

[52] Jiménez M.D.L.V.M., Fernández Domínguez S. (2019). Uso problemático de internet en adolescentes españoles y su relación con autoestima e impulsividad (Problematic internet use in Spanish adolescents and their relationship with self-esteem and impulsivity). Av. Psicol. Latinoam..

[53] Amichai-Hamburger Y., Vinitzky G. (2010). Social network use and personality. Comput. Hum. Behav..

[54] Marino C., Vieno A., Pastore M., Albery I.P., Frings D., Spada M.M. (2016). Modeling the contribution of personality, social identity and social norms to problematic Facebook use in adolescents. Addict. Behav..

[55] Tang J.-H., Chen M.-C., Yang C.-Y., Chung T.-Y., Lee Y.-A. (2016). Personality traits, interpersonal relationships, online social support, and Facebook addiction. Telemat. Inform..

[56] Declaration of Helsinki (2013). Ethical principles for medical research involving human subjects. J. Am. Med. Assoc..

[57] Ortega-Ruiz R., Del Rey R., Casas J.A. (2016). Evaluar el bullying y el cyberbullying validación española del EBIP-Q y del ECIP-Q (Assessing bullying and cyberbullying: Spanish validation of EBIPQ and ECIPQ). Psicol. Educ..

[58] Del Rey R., Casas J.A., Ortega-Ruiz R., Schultze-Krumbholz A., Scheithauer H., Smith P., Thompson F., Barkoukis V., Tsorbatzoudis H., Brighi A. (2015). Structural validation and cross-cultural robustness of the European Cyberbullying Intervention Project Questionnaire. Comput. Hum. Behav..

[59] Puerta-Cortés D.X., Carbonell X., Chamarro A. (2012). Análisis de las propiedades psicométricas de la versión en español del Internet Addiction Test (Analysis of the psychometric properties of the Spanish version of Internet Addiction Test). Trastor. Adict..

[60] Young K.S. (1998). Internet addiction: The emergence of a new clinical disorder. Cyberpsychol. Behav..

[61] Lopez-Fernandez O. (2017). Short version of the Smartphone Addiction Scale adapted to Spanish and French: Towards a cross-cultural research in problematic mobile phone use. Addict. Behav..

[62] Kwon M., Kim D.-J., Cho H., Yang S. (2013). The smartphone addiction scale: Development and validation of a short version for adolescents. PLoS ONE.

[63] Hawi N.S., Samaha M. (2017). The relations among social media addiction, self-esteem, and life satisfaction in university students. Soc. Sci. Comput. Rev..

[64] Beléndez M., Gómez A., López S., Topa G. (2018). Psychometric properties of the Spanish version of the Core Self-Evaluations Scale (CSES-SP). Pers. Individ. Differ..

[65] Hayes A.F. (2018). Introduction to Mediation, Moderation, and Conditional Process Analysis. A Regression-Based Approach.

[66] Longobardi C., Settanni M., Fabris M.A., Marengo D. (2020). Follow or be followed: Exploring the links between Instagram popularity, social media addiction, cyber victimization, and subjective happiness in Italian adolescents. Child. Youth Serv. Rev..

[67] Ferris D.L., Rosen C.R., Johnson R.E., Brown D.J., Risavy S.D., Heller D. (2011). Approach or avoidance (or both?): Integrating core self-evaluations within an approach/avoidance framework. Pers. Psychol..

[68] Song G., Kong F., Jin W. (2013). Mediating effects of core self-evaluations on the relationship between social support and life satisfaction. Soc. Indic. Res..

[69] Sánchez-Martínez M., Otero A. (2009). Factors associated with cell phone use in adolescents in the community of Madrid (Spain). Cyberpsychol. Behav..

[70] De-Sola Gutiérrez J., Rodríguez de Fonseca F., Rubio G. (2016). Cell-phone addiction: A review. Front. Psychiatry.

[71] Bányai F., Zsila Á., Király O., Maraz A., Elekes Z., Griffiths M.D., Andreassen C.S., Demetrovics Z. (2017). Problematic social media use: Results from a large-scale nationally representative adolescent sample. PLoS ONE.

[72] Krasnova H., Veltri N.F., Eling N., Buxmann P. (2017). Why men and women continue to use social networking sites: The role of gender differences. J. Strat. Inf. Syst..

